# Management of patients with SARS-CoV-2 infections with focus on patients with chronic lung diseases (as of 10 January 2022): Updated statement of the Austrian Society of Pneumology (ASP)

**DOI:** 10.1007/s00508-022-02018-x

**Published:** 2022-04-21

**Authors:** Horst Olschewski, Ernst Eber, Brigitte Bucher, Klaus Hackner, Sabin Handzhiev, Konrad Hoetzenecker, Marco Idzko, Walter Klepetko, Gabor Kovacs, Bernd Lamprecht, Judith Löffler-Ragg, Michael Meilinger, Alexander Müller, Christian Prior, Otmar Schindler, Helmut Täubl, Angela Zacharasiewicz, Ralf Harun Zwick, Britt-Madelaine Arns, Josef Bolitschek, Katharina Cima, Elisabeth Gingrich, Maximilian Hochmair, Fritz Horak, Peter Jaksch, Roland Kropfmüller, Andreas Pfleger, Bernhard Puchner, Christoph Puelacher, Patricia Rodriguez, Helmut J. F. Salzer, Peter Schenk, Ingrid Stelzmüller, Volker Strenger, Matthias Urban, Marlies Wagner, Franz Wimberger, Holger Flick

**Affiliations:** 1 Division of Pulmonology, Department of Internal Medicine, Medical University of Graz, Graz, Austria; 2 Division of Paediatric Pulmonology and Allergology, Department of Paediatrics and Adolescent Medicine, Medical University of Graz, Graz, Austria; 3 Department of Pulmonology, Tirol Kliniken, Hospital Hochzirl-Natters, Natters, Austria; 4 Department of Pneumology, University Hospital Krems, Karl Landsteiner University of Health Sciences, Krems, Austria; 5 Department of Thoracic Surgery, Medical University of Vienna, Vienna, Austria; 6 Division of Pulmonology, Department of Medicine II, Medical University of Vienna, Vienna, Austria; 7 Department of Pulmonology, Faculty of Medicine, Johannes-Kepler-University, Linz, Austria; 8 Pulmonology, Department of Internal Medicine II, Medical University of Innsbruck, Innsbruck, Austria; 9 Department of Internal Medicine and Pulmonology, Klinik Floridsdorf, Vienna, Austria; 10 Department of Physical Medicine and Rehabilitation, Klinik Floridsdorf, Vienna, Austria; 11 Specialist for pulmonology, Innsbruck, Austria; 12 Department of Internal and Respiratory Medicine, Hospital Graz II, Hospital Enzenbach, Gratwein, Austria; 13 Department of Pediatric and Adolescent Medicine, Klinik Ottakring, Vienna, Austria; 14 Outpatient Pulmonary Rehabilitation, Therme Wien Med, Vienna, Austria; 15 First Medical Department, Hanusch Hospital, Vienna, Austria; 16 Department of Pneumology, Ordensklinikum Linz Elisabethinen Hospital, Linz, Austria; 17 Specialist for pulmonology, Vienna, Austria; 18 Department of Respiratory and Critical Care Medicine, Karl Landsteiner Institute of Lung Research and Pulmonary Oncology, Klinik Floridsdorf, Vienna, Austria; 19 Allergy Center Vienna West, Vienna, Austria; 20 Department of Pulmonology, Reha Zentrum Münster, Münster, Austria; 21 Interdisciplinary sleep laboratory, Telfs, Austria; 22 Department of Pulmonology, Landesklinikum Hochegg, Grimmenstein, Austria; 23 Specialist for pulmonology, Salzburg, Austria; 24 Department of Internal Medicine and Pulmonology, Klinik Floridsdorf, Vienna, Austria; 25 Ludwig Boltzmann Institute for Lung Vascular Research, Graz, Austria

**Keywords:** Mechanical ventilation, Immune modulators, Chronic lung disease, Pediatric lung disease, Long covid

## Abstract

The Austrian Society of Pneumology (ASP) launched a first statement on severe acute respiratory syndrome coronavirus 2 (SARS-CoV-2) infection in May 2020, at a time when in Austria 285 people had died from this disease and vaccinations were not available. Lockdown and social distancing were the only available measures to prevent more infections and the breakdown of the health system. Meanwhile, in Austria over 13,000 patients have died in association with a SARS-CoV‑2 infection and coronavirus disease 2019 (COVID-19) was among the most common causes of death; however, SARS-CoV‑2 has been mutating all the time and currently, most patients have been affected by the delta variant where the vaccination is very effective but the omicron variant is rapidly rising and becoming predominant. Particularly in children and young adults, where the vaccination rate is low, the omicron variant is expected to spread very fast. This poses a particular threat to unvaccinated people who are at elevated risk of severe COVID-19 disease but also to people with an active vaccination. There are few publications that comprehensively addressed the special issues with SARS-CoV‑2 infection in patients with chronic lung diseases. These were the reasons for this updated statement. Pulmonologists care for many patients with an elevated risk of death in case of COVID-19 but also for patients that might be at an elevated risk of vaccination reactions or vaccination failure. In addition, lung function tests, bronchoscopy, respiratory physiotherapy and training therapy may put both patients and health professionals at an increased risk of infection. The working circles of the ASP have provided statements concerning these risks and how to avoid risks for the patients.

## Epidemiology

In Austria, by 10 January 2022, 13,408 people have died from COVID-19. In the year 2020, COVID-19 accounted for 7% of all deaths. The death rate was strongly associated with age and 97% of all deaths were seen in people >60 years old, while only 0.9% were seen in people <40 years old. Interestingly, if people up to age 70 years are considered, twice as many men as women have died (Statistik Austria), although the infection rate between men and women was not significantly different. The cause for this strong gender effect is unknown.

The most important independent risk factors for death from COVID-19 are obesity, hypertension and diabetes mellitus. In addition, genome-wide association analysis identified blood group A as an independent risk factor (odds ratio [OR] 1.45) and blood group O as a protective factor (OR 0.65) [1]. There had been some controversies about the risk of smoking. Some analyses even considered a protective effect. A recent meta-analysis viewing 1248 publications and including 40 relevant papers, concluded that both former and current smoking significantly increases the risk for COVID-19 death (OR = 1.35 and 2.58, respectively) [2].

## Definitions and course of disease

**Fig. 1:**
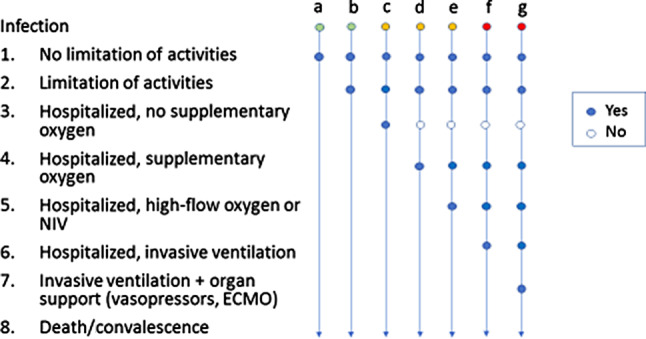
Heterogeneity of SARS-CoV‑2 infection. Infection may be oligosymptomatic (*a,* *b*), or lead to hospitalization with mild disease (*c,* *d*), or severe disease (*e,* *f,* *g*). Each disease course (*a–g*) may result in either death or convalescence, but courses *f* and *g* (*red*) are associated with the highest death rates. *ECMO* extracorporeal membrane oxygenation, *SARS-CoV‑2* severe acute respiratory syndrome-corona virus-2. (8-point scale from WHO R&D blueprint [3])

After infection with the SARS-CoV‑2 virus, there is an incubation time where the virus replicates in the body without causing any symptoms. This used to be about 6 days with previous SARS-CoV‑2 variants, but with the delta variant, the time has decreased to 4 days (reliable data for the omicron variant are pending). As depicted in Fig. 1, after this time infected people may stay nearly asymptomatic (most children and young adults) or develop mild to moderate symptoms that resolve within 4 weeks, or, after another week, they develop a decrease in oxygen saturation <94% with more or less severe respiratory symptoms. This defines a potentially life-threatening course of the disease and applies to a maximum of 2–5% of the infected people in the population. In people with pre-existing hypoxemia, the cut-off at 94% for severe COVID-19 does not apply. Instead, a drop in the oxygen saturation by 4% with no other explanation is considered significant.

If bilateral infiltrates are seen in the chest X‑ray or in the chest computed tomography (CT), the definition of COVID-19 pneumonia is met. If additionally the oxygenation index (OI = PO2/FiO2 [arterial partial pressure of oxygen/inspiratory fraction of oxygen]) is <300, the definition of COVID-19 ARDS is met, provided that cardiogenic lung edema or general fluid retention have been excluded and a positive end-expiratory pressure of 5 mm Hg has been applied. Such patients may need supplementary oxygen, high-flow nasal oxygen, non-invasive ventilation or mechanical ventilation, vasopressive therapy, extracorporeal membrane oxygenation (ECMO) or even lung transplantation.

If the COVID-19 symptoms persist for more than 4 weeks, the definition of long COVID is met (see below). According to a study from Stanford University, 72.5% of all people with symptomatic and mostly hospitalized COVID-19 patients suffered from at least one typical symptom for more than 4 weeks [4]. There is an actual S1 recommendation on long COVID and post-COVID syndrome in the German language by several Austrian and German medical societies including the ASP [5].

## Mechanism of transmission of SARS-CoV-2

For some time, there was controversy about the main mechanism of SARS-CoV‑2 transmission from human to human. Although smear infection appeared unlikely, it was not completely excluded. Transmission over the airways was acknowledged as the main mechanism; however, there was a controversy about the question if only large expelled droplets contained enough virus particles or if also aerosols could cause an infection. Aerosols consist of finest droplets in the range of 0.1–0.5 µm, which are exhaled during every single breath and hover in the room, comparable to cigarette smoke. By 7 May 2021, the Center for Disease Control (CDC) announced that COVID-19 belongs to the airborne infections, meaning that it is effectively transmitted through aerosols. This had already been postulated after analysis of outbreaks and indoor air samples in Wuhan [6] and experimentally proven in an animal model [7]. The aerosol characteristics explain why nearly all infections have taken place indoors, why hand disinfection, face shields and face masks did not provide full protection and why it is of utmost importance to ventilate the rooms where COVID-19 patients stay or have stayed [8, 9].

## Measures to minimize SARS-CoV-2 transmission

When it is unknown if infected people are present, direct contact between people should be avoided. Contamination with large droplets can be prevented by means of face shields and masks and by keeping a distance of 2 m. Aerosol transmission in closed rooms, however, can only be avoided by ventilation of the room as recommended in a WHO document from 2009 [9]. As a minimum ventilation, the room air should be exchanged 4 times per hour. With 12 exchanges per hour, transmissions become very unlikely [9].

It is important that infected people are identified early and that spreading of the virus is prevented. Self-isolation is an adequate method for this purpose. Unfortunately, virus shedding is at its maximum before the first symptoms arise and after COVID-19 recovery, the time until the virus is no longer found in the nose or throat, differs very much between people and can take more than 1 month. Therefore, only a negative PCR test or a high cycle number >30 in the PCR is believed to indicate that there is no significant transmission risk left. Unfortunately, the cut-off for the cycle number depends on the test system (range 24–42 cycles) and systematic investigations on the virus load of the aerosols of infected patients are missing. In the recovery phase of an infection, the PCR tests may show ups and downs of the viral load of the swabs. Therefore, two negative tests, on two different days are necessary before the isolation measures are lifted.

If infected people need help by others, as is usual in the hospital setting, and close contact cannot be avoided, both the patient and the helper need face masks and the room should be well ventilated, with at least 4 air exchanges per hour.

## SARS-CoV-2 mutations

Coronavirus species tend to mutate. Most mutations are meaningless, as they provide no advantage to the virus; however, if a certain mutation spreads faster than others, and causes new outbreaks, it is called a “new variant”. According to a recent convention, the variants are numbered by means of the Greek alphabet. The delta variant has been causing the fourth COVID wave in Austria with a considerable number of deaths, although much lower as compared to the second wave. Currently the omicron variant is spreading with very high speed in many countries like the UK, the Netherlands, and the USA, and will also soon be the leading cause of new infections in Austria. The replication of this variant in the body appears to be much faster than in previous variants explaining higher virus shedding and infection rates and shorter incubation times (https://assets.publishing.service.gov.uk/government/uploads/system/uploads/attachment_data/file/1041896/15-december-2021-risk-assessment-for-SARS_Omicron_VOC-21NOV-01_B.1.1.529.pdf). Unfortunately, the approved mRNA vaccines [10] and the viral vector vaccine ChAdOx1 from Astra Zeneca appear to provide incomplete protection from the omicron variant. It appears that omicron causes a milder course of disease; however, data are still not considered robust enough for a clear statement.

## Pathophysiology

Although a number of molecular and cellular mechanisms of COVID-19 are clarified, many questions remain open. Coronavirus species have been known to account for about 15% of the rhinitis cases over recent years; however, the death rate has been very low. The analysis of the 2002–2004 outbreak due to SARS-CoV‑1 with a high lethality, showed that SARS-CoV‑1, as compared to previous species, was able to enter the cells of the recipient via the ACE2 (angiotensin converting enzyme 2) receptor. SARS-CoV‑2 also binds to the ACE2 receptor, and this might represent an important mechanism contributing to the high lethality; however, there are alternative mechanisms of the virus to enter the cells and in the lungs, and there is a disconnect between the number of ACE2 expressing cells and the number of virus copies within the cellular landscape of the lungs [11, 12]. Moreover, there is no plausible hypothesis to explain why binding of SARS-CoV‑2 to the ACE2 receptor should cause a disease that is characterized by severe endothelial dysfunction and even endothelial structural damage as shown by electron microscopy [13].

**Fig. 2:**
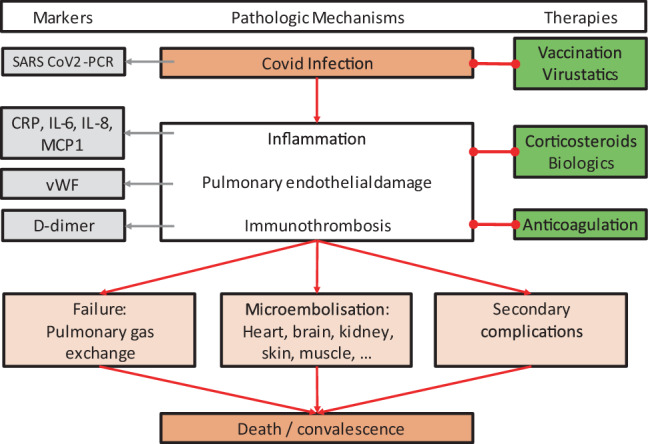
Pathophysiology of COVID-19 ARDS. In the development of SARS-CoV2 infection, acute respiratory distress syndrome (ARDS) results from three major mechanisms, inflammation, endothelial damage and immunothrombosis [14–16]. This causes failure of gas exchange and systemic microembolisms that may mimic vasculitis [17]. *CRP* C-reactive protein, *IL‑6 and IL‑8* interleukin 6 and 8, *MCP1* monocyte chemoattractant protein 1, *ACE2R* angiotensin converting enzyme 2 receptor, *eNOS* endothelial NO synthase

As depicted in Fig. 2, in COVID-19 ARDS patients, there is always a strong involvement of the pulmonary endothelium, which can be induced in cultured human endothelial cells by circulating factors of COVID-19 ARDS patients [14]. The pathological mechanisms may involve immunothrombosis, characterized by the involvement of monocytes, neutrophils and platelets together with a large number of other factors including v. Willebrand factor and IL‑8 [15, 16]. It is likely, that the embolization of pulmonary venous immunothrombosis into the systemic circulation causes disseminated microvascular infarction in all organs, partly mimicking systemic vasculitis [17].

## Symptoms of COVID-19 ARDS

The only specific sign of COVID-19 ARDS is hypoxemia. Unfortunately, due to hyperventilation with decreased pCO2 values, many patients do not suffer dyspnea despite significant and maybe even life-threatening deoxygenation [18]. This mechanism corresponds to the early experiences with hot-air balloons at very high altitude where healthy people died from hypoxemia without feeling dyspnea [19]. Therefore, infected persons with a risk for severe disease should be equipped with a pulse oximeter and instructed in its use if they are outpatients and pulse oximetry should be regularly performed in inpatients with COVID-19 disease.

## Management of COVID-19 ARDS

Patients with COVID-19 ARDS should be hospitalized until unequivocal improvement of findings and symptoms. In hospital, they will be monitored for respiratory decompensation, other infections (pneumonia, urinary tract infection etc.) and thromboembolic events.

**Fig. 3:**
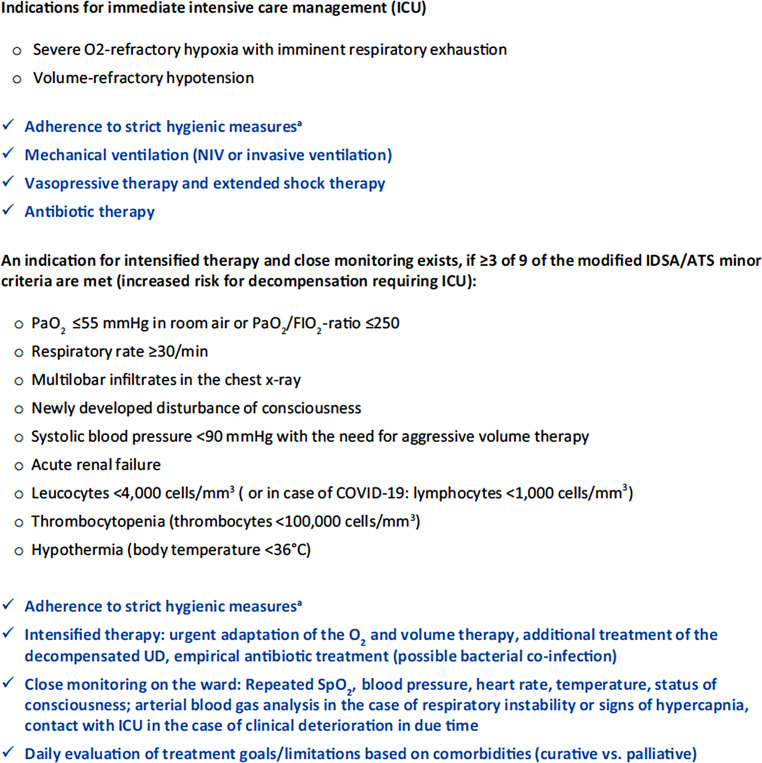
Guidance for the identification of critically ill CAP patients during the COVID-19 pandemic (CAP as an emergency). *CAP* community acquired pneumonia. (From Flick et al. 2020 [20])

**Fig. 4:**
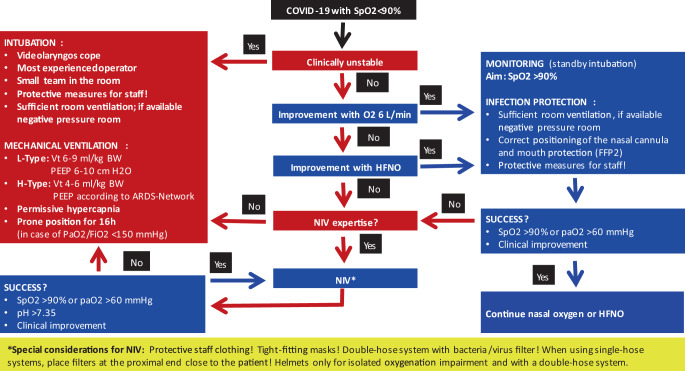
Guidance for the respiratory management of severe SARS-CoV‑2 ARDS. (Adapted from Flick et al. 2020 [20])

As depicted in Figs. 3 and 4, our decision points and algorithm show, when patients should receive nasal oxygen, high-flow nasal oxygen, non-invasive ventilation via nasal of face mask or hood, or invasive ventilation, depending on signs and symptoms, particularly related to oxygenation and breathing rate.

### Anticoagulation

As depicted in Table 1, in hospitalized patients with non-critical COVID-19, therapeutic anticoagulation with LMWH should be considered, except if they have an increased bleeding risk or other contraindications. There is moderate evidence for this after two recent studies showing that therapeutic anticoagulation with LMWH was superior to prophylactic anticoagulation [21, 22]. In both studies the bleeding risk with therapeutic anticoagulation was increased; however, this was not significant and/or outweighed by the beneficial effects of therapeutic LMWH. In the HEP-COVID study, only patients with d‑dimer above 4‑fold upper limit of normal were included [22]. The ATTACC study did not use such a strict inclusion criterion and found less convincing beneficial effects; however, the authors calculated that in 1000 hospitalized patients, therapeutic vs. prophylactic anticoagulation may save 40 lives on the expense of 7 major bleeding events [21]. In contrast, in critically ill COVID-19 patients, therapeutic anticoagulation was inferior to prophylactic anticoagulation due to a higher complication risk. This refers to two studies that found no superiority for therapeutic anticoagulation in terms of the primary endpoint but an increased bleeding rate [22, 23]. In critically ill patients it may be indicated to use half-therapeutic anticoagulation, particularly if patients present with a high venous thromboembolism (VTE) risk profile.

**Table 1:** Anticoagulation in COVID-19 patients

VTE risk					
	For which patient?	Drug	Dose	Comment	Reference
*Standard*	Outpatient	–	–	No evidence for beneficial effects	–
	Hospitalized, non-critical, very high d‑dimer^a^	LMWH/alternatively Fondaparinux	Therapeutic	Superiority vs. prophylactic dose	ATTACC [21] HEP-COVID [22]
	Hospitalized, critically ill	LWWH/alternatively Fondaparinux	Therapeutic	No superiority vs. prophylactic dose; more bleeding	ATTACC [23]
	Hospitalized	LMWH/alternatively Fondaparinux	Half therapeutic	Inferiority vs. prophylactic dose	[24]
*Elevated*	Hospitalized with high VTE risk	LMWH/alternatively Fondaparinux	Half therapeutic	For BMI >35, VTE history, D‑dimer >2 mg/L	[25]
	On ICU	LMWH/alternatively Fondaparinux	Therapeutic	Detrimental effects	[26]
	Acute VTE/ECMO	LMWH/alternatively Fondaparinux	Therapeutic	According to guidelines for acute pulmonary embolism	[27]
	Renal failure	UHF/alternatively Argatroban	As indicated	LMWH not approved	–

*LMWH* low molecular weight heparin, *VTE* venous thromboembolism, *ECMO* extracorporeal membrane oxygenation, *BMI* body mass index, *UHF *unfractionated heparin,* ICU *intensive care unit

^a^The HEP-COVID study enrolled only patients with d‑dimer values above 4‑fold upper limit of normal [22]

If hospitalized patients are discharged early, prophylactic anticoagulation should be continued in the outpatient setting to complete a total time of anticoagulation of minimum 7 days. Anticoagulation for outpatients with COVID-19 is not recommended according to the recent ERS guidelines [28].

### Anti-infective therapy

As depicted in Table 2, antibiotic prophylaxis is not recommended because the rate of opportunistic infections in COVID-19 ARDS is low [29]; however, primary coinfections must be excluded at hospital admission in agreement with the S3 guidelines for community acquired pneumonia [30]. If antibiotic treatment is indicated, the same dose and duration as in patients without COVID-19 should be used. These recommendations have been adapted from the German S3 guidelines [25]. It is important to note that the rate of nosocomial infections in severe COVID-19 ARDS is high, particularly in immunocompromised patients [31] but even in these patients the S3 COVID-19 guidelines did not recommend antibacterial or antifungal prophylaxis but a high level of awareness and routine search for such superinfections.

**Table 2:** Anti-infective therapies in patients with acute SARS-CoV‑2 infection

Anti-infective therapy					
	For which patient?	Drug	Dose and duration	Comment	Reference
–	Primary prophylaxis	Antibiotic or antimycotic or antiviral drugs	–	Negative recommendations because no evidence for benefit	[25, 29]
*Antibiotic/Antifungal therapy*	Primary pulmonary coinfection with bacteria, fungi or other virus	Antibacterial or antimycotic or antiviral drugs	According to CAP guidelines	Treatment according to S3 CAP guidelines 2021	[30]
	Nosocomial infection including aspergillosis	Antibacterial or antimycotic or antiviral drugs	According to HAP and aspergillosis guidelines	Treatment according to HAP guidelines 2018 and aspergillosis guidelines 2021	[32, 33]

Antibiotic and antifungal therapies are only indicated if bacterial or fungal infection is detected but not as prophylactic therapy

*CAP* community acquired pneumonia, *HAP* hospital acquired pneumonia, other infection, e.g. urosepsis

### Immunomodulatory therapy

The main evidence is depicted in Table 3. In an early retrospective study from Wuhan, the application of corticosteroids was associated with a better survival [34]; however, this was also associated with a younger age and higher levels of inflammatory markers. This led to a controversial discussion about the pros and cons of corticosteroids, based on mostly negative studies with corticosteroids in influenza pneumonia. In COVID-19 ARDS, several prospective studies were performed and finally a large randomized controlled open-label study came to the conclusion that 6 mg dexamethasone per day for up to 10 days, improves survival of patients with progressive disease, who need supplemental oxygen or non-invasive or invasive ventilation [35]. The German S3 guidelines [25] and the recent ERS guidelines [28] recommended this treatment in this indication and we do not disagree; however, there were rather negative effects in less severe COVID-19 ARDS and therefore cortisone treatment should not be given to outpatients and those COVID-19 patients who do not deteriorate or need supplemental oxygen.

**Table 3:** Immunomodulatory therapies

Immunomodulatory therapy					
	For which patient?	Drug	Dose	Comment	Reference
*Cortico-steroids*	SpO2 <90%; BF >30/min	Dexamethasone	6 mg/day for 10 days	Dexamethasone vs. control: 28-day mortality −2.8%, HR 0.83, with strong effects in patients on stage 6 (HR 0.64) and moderate effects in stage 4 (HR 0.82)	[25, 35]
	No need for oxygen	Dexamethasone	6 mg/day	Rather detrimental effect in stages 1–3	[25, 35]
*Biologics*	High oxygen need but not on MV	Tocilizumab	Ca. 8 mg/kg BW, max 800 mg, once	EMA approval for patients on oxygen or mechanical ventilation due to COVID-19 who are receiving systemic corticosteroids	[36], INN
	Need for MV	Tocilizumab	Ca. 8 mg/kg BW, max 800 mg	No efficacy signal	[25]
	Bacterial/fungal infection	Tocilizumab	–	Contraindication	–
	Need for oxygen with high risk for mechanical ventilation	Anakinra	100 mg s.c., once	EMA approval for COVID-19 patients on oxygen at high risk of mechanical ventilation who present with suPAR levels ≥6 ng/mL	[37], INN
	<72 h hospitalized, up to stage 4	Tofacitinib	10 mg BID up to 14 days	Beneficial effects, few side effects	[38]
	Stage 4–7	Baricitinib	4 mg OD	High quality studies with significant beneficial effects on important endpoints. EMA application for COVID-19 patients on oxygen	[39–42], EMA homepage

Stages 1–3, 4 and 6 relate to 8‑point scale from WHO R&D blueprint [3]. Compare Fig. 1!

*BF* breathing frequency, *MV* mechanical ventilation, *HR* hazard ratio, *EMA* European Medical Agencies, *suPAR* soluble urokinase-type plasminogen activator receptor, *BID* bis in die, i.e. twice daily, *OD* once daily

Tocilizumab, an interleukin‑6 (IL-6) antagonist approved for rheumatoid arthritis, is of particular importance because it received conditional recommendations by the German S3 [25] and the ERS guidelines [28]. The ASP has evaluated the evidence and commented these guidelines (Flick et al. Positionspapier-der-OeGP-zu-aIL-6R-bei-COVID_final_Mai2021_IC.pdf [ogp.at]). There were two high-quality randomized controlled blinded studies which did not suggest improved mortality with tocilizumab as compared to placebo [43, 44] and there was a subgroup of the RECOVERY study where 4116 patients were 2:1 randomized to tocilizumab and usual care. In this latter study, the mortality was in favor of tocilizumab (OR = 0.85), relatively homogeneously distributed over all subgroups. Also, several secondary endpoints were in favor of tocilizumab [36]. Of note, this was an open-label platform trial (not the highest quality standard) and patients were only eligible if CRP was ≥75 mg/L and if there was no uncontrolled bacterial or fungal infection. Based on these data, we agree with the S3 and the ERS guidelines to give a conditional recommendation for the use of tocilizumab (Table 3).

Anakinra, an interleukin‑1 (IL-1) antagonist approved for rheumatoid arthritis, is also approved for COVID-19 by the EMA. According to the Committee for Medicinal Products for Human Use (CHMP) review, a study involving 606 hospitalized adults with moderate or severe COVID-19 pneumonia who had soluble urokinase plasminogen activator receptor (suPAR) levels of at least 6 ng/ml showed that anakinra was effective at treating COVID-19 [45–47]. These patients received anakinra or placebo in addition to standard of care. Standard of care for most patients included low or high-flow oxygen and dexamethasone, and some also received remdesivir. The study showed greater clinical symptom improvements in patients treated with anakinra plus standard of care compared with those who received placebo plus standard of care. Anakinra reduced the risk of a patient’s condition worsening to more severe disease or death during the 28-day study period compared with placebo. The treatment benefit of anakinra compared to placebo was supported by an increase in the number of patients who fully recovered and a reduction in the number of patients whose condition worsened to severe respiratory failure or death; however, as the suPAR biomarker is not widely available, this treatment concept is difficult to apply in clinical practice.

Baricitinib, a Janus kinase inhibitor approved for rheumatoid arthritis and atopic dermatitis, has been tested in two phase III randomized placebo-controlled trials. In the first trial with 1033 patients, there was a significant beneficial effect on recovery time [39], in the other trial with 1525 patients, there was a significant reduction of mortality with baricitinib as compared to placebo (HR 0.62, *p* = 0.005) [40]. The quality of both trials was high and beneficial effects appeared substantial as compared to the adverse effects. Application for approval was submitted at EMA. The CHMP is in the review process for the indication COVID-19 ARDS with need for low-flow or high-flow oxygen (Table 3).

There have been a large number of studies using other repurposed drugs to treat COVID-19. The WHO Solidarity Trial, a parallel group randomized controlled open-label trial, investigated remdesivir, lopinavir, interferon, and hydroxychloroquine and found no significant beneficial effects of any drug on mortality, initiation of mechanical ventilation or hospital duration [48]. There were multiple other studies on other drugs and vitamin D3, which are summarized in Table 3. All the respective recommendations of the German S3 guidelines have been negative [25].

### Antiviral therapies

Two major approaches have been applied to cause direct antiviral effects: small interfering molecules with virustatic properties like remdesivir, molnupiravir and PF-07321332/ritonavir on the one hand and serum from convalescent COVID-19 patients or monoclonal antibodies against the spike protein. Except for convalescent plasma, the other approaches have been EMA approved or they are in review. All successful studies have been performed in unvaccinated patients with initiation of therapy in the first days after start of symptoms (Table 4).

**Table 4:** Antiviral therapies

Antiviral therapy					
	For which patient?	Drug	Dose	Comment	References
*Virustatics*	Pre-hospital or need for oxygen or non-invasive but not invasive ventilation	Remdesivir (Veklury™)	I.v. infusions for 3 days	EMA approval based on one study with hospitalized and one study with pre-hospital patients	[49, 50], INN
	Positive COVID-19 test results with no need for oxygen who are at high risk for progression to severe COVID-19	Molnupiravir (Lagevrio™)	4 caps twice daily (800 mg) BID for 5 days	UK approval. EMA application. Can already be prescribed	[51], EMA homepage
	Positive COVID-19 test results with mild-to-moderate disease who are at high risk for progression to severe COVID-19	Nirmatrelvir/ritonavir (Paxlovid™)	1 caps of each drug BID for 5 days	FDA approval. EMA application. Can already be prescribed	EMA homepage
*SARS-CoV‑2 antibodies*	Moderate to severe ARDS	Bamlamivimab	–	No efficacy signal	[52]
	–	LY-CoV555	n. a.	Only surrogate endpoints	[53]
	Positive COVID-19 test results with no need for oxygen who are at high risk for progression to severe COVID-19	Casirivimab + imdevimab (Ronapreve™)	1200 mg s.c. once	Preventive approach. FDA approval and EMA approval	[54, 55], INN
	Positive COVID-19 test results with no need for oxygen who are at high risk for progression to severe COVID-19	Sotrovimab (Xevudy™)	500 mg iv once	Preventive approach. FDA and EMA approval	INN
	Positive COVID-19 test results with no need for oxygen who are at high risk for progression to severe COVID-19	Regdanvimab (Regkirona™)	40 mg/KG i.v. once	EMA approved	INN
	–	Convalescent plasma	–	Negative results	[56–58]
*Other*	–	Azithromycin	–	Negative results	[59–62]
	–	Ivermectin	–	Negative results	[63–65]
	–	Vitamin D 3	–	Negative results	[66, 67]
	–	Interferon beta	–	Negative results	[68]
	Post exposition and all tested stages	Chloroquin/hydroxy-chloroquin	–	Negative results	[48, 69–72]

With reference to AWMF S3 guidelines [25]

*INN* international nonproprietary name: official information on EMA-approved drugs

Remdesivir is applied as intravenous infusion for 3 days and received EMA approval after a study in hospitalized COVID-19 patients [49] and a study in non-hospitalized patients at risk of a severe disease [50]; however, as already mentioned, in the Solidarity Trial, remdesivir did not reduce the mortality rate [48]. Molnupiravir (Lagevrio™) is applied as 4 capsules (800 mg) BID for 5 days and showed a significant benefit in the combined endpoint, hospitalization or death at day 29 with 6.8% vs. 9.7% in the drug vs. placebo group. The most impressive result was the reduced death rate with 1 vs. 9 patients [51]. The EMA is currently evaluating the marketing authorization application; however, this medicine can already be used in the EU to treat COVID-19, after EMA’s CHMP completed its review. For Paxlovid™, a combination of PF-07321332 (nirmatrelvir) and ritonavir, EMA has started a rolling review process after a prospective study, showing a highly significant benefit in the combined endpoint of hospitalization or death at day 28, which was met by 1% vs. 7%. The drug comes in capsules and is taken BID for 5 days. It has been made available to the US population by the Biden administration and it can also be prescribed in Europe (positive CHMP assessment report and authorization expected). Convalescent plasma from patients with a recent SARS-CoV‑2 infection showed no effects in severe (late) COVID-19 ARDS. Specific antibodies against the SARS CoV‑2 spike protein showed significant beneficial effects; however, only in very early and mostly prehospital patients (preventive therapy). The combination of casirivimab and imdevimab (Ronapreve™) has been approved by the FDA as Regen-Cov™ and is now also approved by the EMA after positive studies [54]. Regdanvimab (Regkirona™) is also approved by the EMA. It was developed by the South Korean company Celltrion and is manufactured in Hungary. It was tested in a large prospective randomized study enrolling 1315 patients (80% European) and met its primary endpoint, rate of death, hospitalization or need of oxygen at day 28 with 3% vs. 11% in drug vs. placebo patients. It is easily available in Austria. It is given as single i.v. infusion of diluted drug at a dose of 40 mg/kg. Sotrovimab (Xevudy™) has also been approved by the EMA. It employed a combined primary endpoint of death or hospitalization after 28 days in a study with 1057 patients, where the endpoint was met by 1% vs. 6%. It is given intravenously for 5 days. Evusheld™, a combination of tixagevimab and cilgavimab, is currently in rolling review by the EMA and not yet available.

In conclusion, virustatic therapies are very attractive; however, they are not arguments against vaccination. From a practical point of view, oral drugs (molnupiravir and PF-07321332/ritonavir) are most important, because they are needed by non-hospitalized patients who have no or mild symptoms and have just been tested positive for COVID-19. All other drugs need intravenous infusions which is more difficult in the outpatient setting.

### Intubation vs. non-invasive ventilatory support (NIV)

The German S3 guidelines [25] recommend that an oxygenation index (OI) <150 mm Hg represents a conditional indication for intubation and invasive ventilation and OI <100 mm Hg represents an urgent indication. We agree with this recommendation and provide an algorithm for the hospitalized patient (Fig. 4). The advantage of intubation is that by means of filters in the expiratory tube, there is no further contamination of the room with virus-laden aerosols; however, reports from China and the USA reported mortality rates of 60–80% for patients who were intubated and mechanically ventilated. The advantage of non-invasive ventilation is that the patient does not need deep sedation, which is associated with much lower risk of complications. The rationale to first try non-invasive ventilation before intubation is discussed in detail by Windisch et al. [73, 74]. The actual German data confirm the published mortality of 22% for patients with ventilatory support [25, 75]; however, the mortality depends very much on the setting in which mechanical ventilation is started (particularly outside the ICU), the clinical condition of the patient and the criteria for admission to the ICU. Finally, the indication for intubation is always based on clinical judgement (exhaustion, ventilatory drive, level of consciousness). It is very important to consider palliative care or terminal comfort care in individual patients, based on fragility, comorbidities or patients’ preferences. There have been positive experiences with inclusion of palliative care specialists in these difficult decisions [76].

### Extracorporeal membrane oxygenation and lung transplantation for COVID-19 ARDS

In patients with progredient respiratory failure despite optimized mechanical ventilation, including prone position, it may be indicated to initiate ECMO. This avoids secondary damage to the lungs by high oxygen levels and high ventilatory forces; however, it is an unsolved question which patient is the right candidate for this treatment [77].

Even more challenging is the question who to consider for lung transplantation. There is a broad consensus that lung transplantation should only be considered in COVID-19 ARDS patients with irreversible lung damage, who fail to regenerate their native lungs despite several weeks of ECMO [78]. Criteria to select eligible ARDS patients for lung transplantation include: i) a negative virus status (virus culture or PCR), ii) no clinical/radiological improvement despite MV and/or ECMO support, iii) mono-organ failure, iv) absence of severe extrapulmonary comorbidities and v) a realistic potential for long-term recovery [79, 80]. The time period necessary to determine native lung recovery has been a moving target throughout the pandemic. Currently, a period of at least 6 weeks on ECMO is recommended before a patient can be listed for lung transplantation; however, a considerable number of COVID-19 ARDS patients develop recurrent bacterial superinfections/bacteremia, necrosis of large parts of the lungs, severe pulmonary or pleural hemorrhage or recurrent tension pneumothorax despite large bore drainage. All these clinical scenarios might warrant an earlier transplantation [81].

To date 22 COVID-19 ARDS patients have received a lung transplantation in Austria, nearly all had a favorable short-term outcome. The majority of these patients could be successfully discharged from hospital; however, data on long-term results are not yet available.

## Vaccination for SARS-CoV-2

Vaccination is by far the most effective measure to prevent severe COVID-19 and death. According to AGES, actually 90% of all COVID-19 cases are unvaccinated persons (https://www.ages.at/themen/krankheitserreger/coronavirus/; by 23.10.2021). In Austria, 4 vaccines have been marketed: mRNA vaccines by Biontech/Pfizer, and Moderna, and viral vector vaccines by Janssen and Astra Zeneca. Recently, a protein-based vaccine, NVX-CoV2373 by Novavax, has been recently approved by the EMA but has not received market authorization in Austria. The CDC provides the actual information on efficacy and adverse effects (https://www.cdc.gov/coronavirus/2019-ncov/vaccines/Your COVID-19 Vaccination | CDC). All vaccines are efficacious, although the Astra Zeneca vaccine showed no sufficient efficacy in the South African variant. All products need 2 intramuscular injections, 3–8 weeks apart. The Janssen product was designed for a single vaccination; however, this appears to provide less protection as compared to the other vaccines with 2 injections. Recent data suggest that the Biontech/Pfizer vaccine is safe and very effective in children ≥12 years old [82] and that it may be safe and effective in pregnant women [83].

Actually, there is much discussion about breakthrough infections. A recent study showed that with the Biontech/Pfizer vaccine, such infections were rare (2/417) and due to new variants [84]; however, the actual rates of vaccinated people with new SARS-CoV‑2 infections have significantly increased and by October 2021 reached 10% in Austria (https://www.ages.at/themen/krankheitserreger/coronavirus/; by 23.10.2021). This has led to ‘Anwendungsempfehlungen des Nationalen Impfgremiums’ of the Austrian government (by 17.08.2021), recommending off-label vaccination of people who have received a first vaccination 6–9 months before and have an increased risk for breakthrough infections or who are working in the healthcare system (https://oegit.eu/wp-content/uploads/2021/08/COVID-19-Impfungen: Anwendungsempfehlungen des Nationalen Impfgremiums (oegit.eu).

Adverse effects in the first 2 days after vaccination are very common and include fever, muscle pain and headache, but do not cause harm. Serious adverse effects have developed later and they are very rare. The Centers of Disease Control and Prevention (CDC) posted to following warnings: “CDC has received increased reports of myocarditis and pericarditis in adolescents and young adults after COVID-19 vaccination. The known and potential benefits of COVID-19 vaccination outweigh the known and potential risks, including the possible risk of myocarditis or pericarditis. CDC continues to recommend COVID-19 vaccination for anyone 12 years of age and older. Johnson & Johnson’s Janssen (J&J/Janssen) COVID-19 Vaccine: CDC and the FDA recommended that use of (J&J/Janssen) COVID-19 Vaccine resume in the United States, effective April 23, 2021. However, women younger than 50 years old should especially be aware of the rare risk of blood clots with low platelets after vaccination. There are other COVID-19 vaccines available for which this risk has not been seen.”

The Astra Zeneca vaccine has also been associated with rare blood clots (cerebral vein thrombosis) and with thrombocytopenia, which is caused by autoantibodies against platelet factor 4. Greinacher et al. [85] reported 11 patients from Germany and Austria, *n* = 9 female, mean age 36 years (22 years to 49 years old), who had received the Astra Zeneca vaccine 5–16 days before the first symptom, but no heparin. The most common event was cerebral venous thrombosis (*n* = 9) and finally 6/11 patients died. The underlying mechanism appears to be similar to heparin-induced thrombocytopenia [85–88] and has been termed vaccine-induced prothrombotic immune thrombocytopenia (VIPIT) in a guideline statement published by the Society for Thrombosis and Hemostasis Research (GTH) [89]. This statement gives recommendations for diagnosis and treatment of this rare complication, e.g. that the adequate treatment of VIPIT is administration of high-dose immunoglobulins (1 g/kg BW) on 2 consecutive days.

## Pediatric pulmonology

The incidence of SARS-CoV‑2 infection in children and adolescents is lower than in adults (HR 0.56) [90]. Children have lower viral loads than adults [91] which may explain that transmission by children tends to be lower than by adults, although reports about transmission rates are heterogeneous [92]. A study from India suggested that the majority of transmissions from children occur between age 12 and 18 years [93]. Because children are often asymptomatic after SARS-CoV‑2 infection, the number of transmissions from children may be underestimated. There are several hypotheses why children have a lower COVID-19 morbidity: a stronger immune response [94], a different microbiome, lower density of ACE2 receptors and less comorbid conditions [95].

The COVID-19 symptoms are very heterogeneous among children and adolescents ranging from asymptomatic to life-threatening disease. The most frequent symptoms are fever, cough, and gastrointestinal symptoms. The incidence of severe COVID-19 disease in Europe is not known; however, in the USA the rate of infected children admitted to hospital was 1.0–1.3%, to the ICU it was 0.1% and the mortality was far below 0.1% [96]. Adolescents were more affected than small children.

A total of 582 children from a multicenter European network with severe COVID-19 in subjects <18 years old were analyzed. The mean age was 5 years, the m:f ratio was 1.15, with pre-existing medical conditions in 25%. Of these children, 63% were admitted to hospital, 8% to the ICU, and 4% required mechanical ventilation. The overall fatality rate was 0.7% [97]. Among the factors associated with an increased risk for severe COVID-19, concomitant viral infections and lymphopenia were identified [98].

An ERS study based on a questionnaire sent to specialists for pediatric pulmonology, analyzed data from 945 children with pulmonary comorbidities, stratified as asthma, cystic fibrosis (CF), bronchopulmonary dysplasia (BPD), and other diseases. It showed that only BPD and other diseases were associated with severe COVID-19 [99]. There was no evidence that asthma or CF increase the risk for severe COVID-19. There is an online European registry about COVID-19 in CF patients, showing that the COVID-19 mortality among CF patients is small (1459 registered patients, 17 deaths as of 21.05.2021) [100].

In the daily routine in pediatric pulmonology, it is a challenge that there are often false or correct positive SARS-CoV‑2 tests, where the true cause of symptoms is something else. A survey from Graz found just 10 positive cases among 1100 admissions to the pediatric emergency room [101]. Another challenge is that there is no authorization for vaccination in children <12 years.

The most common severe course of COVID-19 in children represents pediatric inflammatory multisystem syndrome, temporally associated with SARS-CoV‑2 (PIMS-TS) or multisystem inflammatory syndrome in children (MIS-C). Both terms have been used synonymously, however, with no homogeneous definitions [102, 103]. In a study of 953 cases, the median age was 8 years, and male gender (59%) and ethnic minorities (black) (37%) were overrepresented. Obesity was found in 25% but other comorbidities were rare. The disease was characterized by fever (100%), gastrointestinal symptoms (85%), cardiocirculatory manifestations (79%), increased inflammatory biomarkers and 50% presented with respiratory symptoms. Over half of patients (56%) presented with shock and the majority (73%) needed intensive care treatment, including ECMO in 4%. Despite severe disease, mortality was low (2%) [103]. Therapies like immunoglobulins, high-dose aspirin, and cortisone have been used. Two recent studies compared immunoglobulins vs. cortisone and the combination of both approaches. It appears that the effects of immunoglobulins and cortisone are equivalent [104] and that the combination might be more effective than immunoglobulins alone [105]. The disease is reminiscent of Kawasaki disease; however, cardiac involvement is more diffuse, is probably caused by a cytokine storm and has a better prognosis [106].

In summary, so far the prevailing impression has been that children suffer more from sequelae of the lock-down, deprivation from education and culture, social isolation, reduced exercise training, prolonged screen-time and the economic consequences of the pandemic than from the disease itself.

## Sleep medicine

The COVID-19 pandemic has affected both insomnia and sleep-associated breathing disorders, like obstructive sleep apnea syndrome (OSAS), central sleep apnea syndrome (CSAS) and the combined forms.

According to a recent meta-analysis [107], the incidence of insomnia has significantly increased during the pandemic. The COVID-19 patients suffer from insomnia in 75% of cases. This may be explained by cough, fever, isolation, fears, and dyspnea. During the pandemic, also health professionals suffered more often from insomnia (36%) than the general population (32%). In long COVID patients (see below), insomnia is one of the severe problems (21%) [108].

The risk for severe COVID-19 and death is increased in sleep apnea [109] because obesity, arterial hypertension and heart failure are important risk factors for both sleep apnea and severe COVID-19. In addition, the CORONADO study showed that in diabetic patients, OSAS is an independent risk factor for severe COVID-19 [110]. This explains why 21–28% of severe COVID-19 patients had also severe sleep apnea syndrome [111, 112].

The OSAS is often treated with a continuous positive airway pressure (CPAP) device which is adapted in a diagnostic and/or therapeutic overnight stay in a sleep laboratory. During the lock-down, in Austria about 80% of the sleep laboratories were closed. This has caused delays in the diagnostics and therapy of the disease. Indeed, CPAP therapy may cause room contamination with virus-loaden aerosols, increasing the risk of contamination of relatives and health personal [113]. This has caused treatment cessation in many cases.

The therapy indications have not changed due to the pandemic but the case management has changed. During the pandemic, telemedicine has become an important tool in the management of patients and this kind of management has considerably improved due to the pandemic. A French study even found that during the pandemic, the compliance with CPAP therapy increased significantly [114].

All patients with insomnia and sleep apnea should get vaccinated against SARS-CoV‑2 and all patients with significant sleep apnea syndrome should continue their therapy during the pandemic and during acute COVID-19. Diagnostics including sleep laboratory, should be continued during the pandemic.

## Asthma

In a large association study from the UK including 17 million NHS primary care patients, over 10,000 COVID-19 deaths were analyzed. There was no significant association between asthma and COVID-19 death, except for patients on oral corticosteroids [115]. Systematic reviews did not detect a clear increase or decrease in risk [116]. Within asthma patients, people with comorbid COPD and people with non-allergic (compared to allergic) asthma appear more vulnerable to worse outcomes and among these there is a male preponderance (64%) and high incidence of smoking (62%) [117].

Inhaled corticosteroids: inhaled corticosteroid (ICS) use does not appear as risk factor for severe COVID-19, although data on medication use is difficult to interpret. One mechanism of virus entry into target cells is binding to the ACE2 receptor (ACR2R) and TMPRSS2 (another receptor). A study analyzing the expression of ACE2R and TMPRSS2 RNA in cells from induced sputum found no differences between asthma patients and healthy controls, however, lowered expressions in asthma patients on high-dose ICS [118]. This could mean that ICS therapy represents a protective factor for severe COVID-19 ARDS. There are two open-label studies from the UK, suggesting protective effects of inhaled budesonide in the early phase of SARS-CoV‑2 infection, independent of asthma [119, 120]; however, these data are difficult to interpret and might be subject to bias. There is a German/Austrian statement recommending the continuation of ICS therapy in asthma patients during COVID-19 (Idzko et al. Glukokortikoide-Covid19-STOIC-Studie_04-2021_IC.pdf (ogp.at)).

Biologicals: a recent analysis from a large Israeli database suggested that biologics are not associated with COVID-19 incidence and outcome and also oral corticosteroids are not associated with COVID-19 incidence; however, with significantly worse outcome [121]. There is a statement of Austrian Society for Allergology and Immunology (ÖGAI) and ASP, recommending the continuation of therapy of severe asthma with biologicals in case of SARS-CoV‑2 infection (https://www.ogp.at/biologika-therapie-und-sars-cov2-covid-19/).

Asthma medications should be maintained or intensified to attain a controlled asthma state. This applies to all asthma medications including ICS and biologics. Oral or parenteral corticosteroids should be avoided in the long-term maintenance therapy.

Vaccination: in a statement of DGP and ÖGP, for patients on biologicals for severe asthma, COVID-19 vaccination has been recommended (https://www.thieme-connect.com/products/ejournals/abstract/10.1055/a-1373-9381). There were few reports of asthma exacerbations in response to COVID-19 vaccination, in uncontrolled asthma, however, the risk of vaccination appears smaller than the benefits. Before vaccination against SARS-CoV‑2, asthma therapy should be evaluated by a pneumologist or experienced general physician and adapted, if necessary.

## COPD

A large NHS association study found an increased COVID-19 mortality for respiratory diseases excluding asthma by 63%, even after correction for all co-variates [115], suggesting that COPD represents an independent risk factor for severe COVID-19. A recent UK biobank analysis including 430,000 patients found that the risk for severe COVID-19 was increased by both COPD (37%) and an intermediate and strong genetic risk (34% and 50%). If COPD and the strong genetic risk were combined, the risk for severe COVID-19 was increased by 105% [122]. The probable causes for increased COVID-19 mortality in COPD patients are pre-existing lung function and diffusion limitation, pulmonary endothelial dysfunction, and possibly elevated lung tissue expression of ACE2 receptor, and other factors that may facilitate virus entry in cells [123, 124]. There was no significant association between inhaled corticosteroid use and COVID-19 severity or death [125]. Interestingly, during the lock-down in the USA, COPD admissions were reduced by half, probably due to reduced seasonal respiratory virus infections [126].

COVID-19 patients with COPD should receive optimized inhaled COPD therapy and we recommend that such patients, independent of stage, are vaccinated against COVID-19.

## Lung cancer

According to the NHS association study, cancer (excluding hematologic malignancies) is associated with an increased COVID-19 mortality after correction for co-variates, by 72% [115]. This may be explained by the fact that many patients suffer from wasting of immunologic defense mechanisms by the disease itself and due to anti-cancer therapies. In addition, reluctance to ICU admission may contribute to this result.

We recommend that chemotherapy, radiotherapy and other immunosuppressive therapy should be halted during acute COVID-19 disease. There is uncertainty about PD-L1 and PD‑1 based therapies. They have been well tolerated [127] but also associated with exaggerated pneumonitis [128].

Cancer patients should be vaccinated against COVID-19 as early as possible. They may need booster vaccinations earlier than other people.

## Interstitial lung disease

Interstitial lung diseases (ILD) comprise many different entities with acute and chronic courses and different therapeutic concepts, depending on the specific diagnosis.

COVID-19 pneumonia may cause long-term and eventually even irreversible ILD. A study from Innsbruck found lung parenchymal abnormalities in 62% of the patients, 12 weeks post-COVID [129]. A prospective follow-up study by Myall et al. found signs of organizing pneumonia in about 5% of cases after 6 weeks, with a remarkable response of DLCO and vital capacity (VC) to a pulsed corticosteroid therapy over 3 weeks starting with 10–35 mg/day [130]. This suggests that such a therapy should be considered if 6 weeks post-COVID the patient presents with functional changes and ground glass opacities or organizing pneumonia in the chest CT; however, the evidence is limited because there was no control group. A recommendation is not possible as no long-term data are available. The role of antifibrotic therapies like pirfenidone and nintedanib to prevent post-COVID-19 ILD is addressed in ongoing studies (NINTECOR and ENDCOV-I).

Patients with interstitial lung disease (ILD) have an increased risk for severe COVID-19 ARDS. According to Esposito et al. COVID-19 patients with ILD vs. matched controls without ILD had a mortality rate of 33% vs. 13% [131]. This was confirmed by Drake et al. who found a HR of 1.6 vs. matched controls. In the most common subgroup, idiopathic pulmonary fibrosis (IPF), the HR was even 1.74 [132]. This may be explained by the fact that ILD patients have pre-existing restriction of the lungs, reduced gas exchange capacity, need for supplemental oxygen, and immunosuppressive treatment. In addition, IPF patients typically present at an advanced age >65 years and are predominantly male.

ILD patients should try everything to avoid a SARS-CoV‑2 infection. This also relates to measures in the clinic where the staff should be immunized (vaccinated and/or recovered from infection). Contacts with other patients should be avoided by adaptation of the time schedule to minimize waiting and contact times. Telemonitoring (videochats) may be helpful to avoid some of the direct contacts.

Practical considerations:

In the case of contact with an infected subject or in case of a deterioration of gas exchange, a SARS-CoV‑2 PCR should be performed without delay. If positive, a preventive treatment (e.g. casirivimab-imdevimab) should be considered. The management of a SARS-CoV‑2 infection in an ILD patient principally agrees to the common recommendations for other patients (Figs. 3 and 4).

A long-term immunosuppressive therapy should be continued as well as an antifibrotic therapy. In the case of a recently started or just planned immunosuppressive therapy, an individual decision is necessary. It is recommended to use a shared decision-making to find the best option for the given situation.

All ILD patients should be vaccinated against SARS-CoV‑2. There is no argument against, because the risks of the disease are far greater than the risks of the vaccination, also in case of immunosuppressive treatment.

## Pulmonary hypertension

In the large COVID-19 databases, pulmonary hypertension (PH) has not been evaluated. A recent PH care survey of 1073 PH patients from 52 countries worldwide found that COVID-19 related events were reported in only 1% of the survey responders; however, 8% reported health deterioration, 4% hospitalization for PH, 11% reported difficulties to access their PH expert center, and 3% interruption of treatment due to shortage of medication. Anxiety or depression was reported by 67% of the participants [133]. This suggests that the lock-down and its associated problems affected the PH community more than COVID-19 itself. A late breaking poster at the recent ERS conference reported about 211 PH patients in France with acute SARS-CoV‑2 infection [134]. The overall mortality was 25%. In the subgroups of patients there were striking differences in the mortality. Female vs. male mortality was 15% vs. 36%, never smokers vs. former smoker mortality was 12% vs. 40%. Mortality was very high among patients with pre-existing lung, heart and/or kidney disease, low DLCO and/or low 6 min walking distance.

None of the PAH therapies have been associated with COVID-19 outcomes. We recommend that patients receive their regular medication and that they are seen in regular intervals in their expert centers.

All PH patients should be vaccinated against SARS-CoV‑2 as soon as possible.

## Post-COVID syndrome/long COVID

The definitions of post-COVID syndrome and long COVID are just related to the time after an acute SARS-CoV‑2 infection and not to any clinical features [135]. We distinguish:

- the acute phase of COVID-19, which may last up to 4 weeks,
- COVID-19 associated symptoms up to 12 weeks after infection, and
- post-COVID syndrome with complaints for more than 12 weeks after infection.

Long COVID is a patient-derived term that includes symptoms lasting for more than 4 weeks.

The pathologic mechanisms underlying long COVID are not known, but it appears they are heterogeneous and multifactorial: 1) persisting SARS-CoV‑2 virus or virus components, 2) tissue damage and 3) persisting inflammation have been discussed in the literature [136].

In daily practice it makes sense to distinguish between symptoms due to inflammation, due to tissue or organ damage and sequelae of hospitalization including sarcopenia and social isolation. For example, pulmonary embolism may cause long-term effects like dyspnea, that have extensively been discussed in the literature. Therefore, in such a case, a new pathological mechanism, specific for COVID-19 appears unlikely.

Simple laboratory investigations may uncover treatable causes, e.g. of fatigue—anemia, vitamin D deficit, hypothyroidism, cortisol deficit, chronic renal insufficiency. A screening for all these diseases should be done in long COVID patients with predominant fatigue.

The S1 guidelines (point of care tool) for diagnostics and therapy of long COVID in cooperation between several Austrian medical societies including ASP, have recently been launched [5].

### Functional scoring of long COVID

Klok et al. have published a post-COVID functional score (PCFS) that has been validated for post-COVID patients [137]. In grade 0, patients are free of symptoms, in grade 1 they have symptoms but can cope with all activities of daily life, in grade 2 they avoid some of these activities due to symptoms, in grade 3 they are unable to do some of these activities, and in grade 4 they are no longer able to cope with daily life and need regular care. This simple scale is suitable for both the initial assessment and the follow-up. For details see [137].

We recommend inviting patients with a PCFS ≥2 to undergo multidisciplinary diagnostics and eventually rehabilitation.

### Rehabilitation for long COVID

The WHO distinguishes 4 phases of rehabilitation:

- Phase 1: mobilization in hospital. In Austria we can choose between outpatient and inpatient rehabilitation
- Phase 2: either 3 weeks inpatient or 6 weeks outpatient rehabilitation
- Phase 3: follow-up treatment, outpatient rehabilitation for 6–12 months for patients with severe limitations
- Phase 4: consolidation of the rehabilitation by integration of the training into daily life

Dyspnea on exertion and/or fatigue belong to the common complaints of long COVID patients. There are already some studies showing safety and remarkable efficacy of cardiopulmonary rehabilitation in this indication [138–140], although randomized controlled studies are missing. Before starting a rehabilitation program, extensive diagnostics are necessary to exclude coronary heart disease, myocarditis, pulmonary embolism, and disturbances of lung function or gas exchange [141]. All these diseases necessitate special diagnostics and treatment and rehabilitation is secondary to that.

Patients with PCFS ≥2 who are able and interested, should be offered ambulatory rehabilitation to reconstitute their functional limitations.

Ambulatory rehabilitation includes:

- cardiorespiratory physiotherapy
- ergotherapy
- psychotherapy, neuropsychology and/or
- speech therapy including swallowing training

If ambulatory therapy is not sufficient, multimodal or inpatient rehabilitation should be considered. This agrees with the German AWMF guidelines [142].

If the pneumologic, neurologic and cardiologic limitations are predominant, a specific rehabilitation should be prescribed.

For neurologic, psychotherapeutic, psychiatric and logopedic rehabilitation we refer to the recent S‑1 guidelines [5].

## Bronchoscopy

Bronchoscopy represents a relatively high risk for contamination of nursing staff and physicians. Both droplets and aerosols may cause contaminations. Other ways of contamination are unlikely if usual hygienic standards are applied.

Practical considerations:

All personnel should be vaccinated against SARS-CoV‑2, FFP‑2 masks should be worn by both personnel and patients, whenever possible.

Room ventilation should be sufficient (at least 4 exchanges per hour) to avoid contamination by aerosols.

## Pulmonary function test

Pulmonary function test, diffusion capacity, blood gas analysis, and exercise tests are done in close contact between technicians and patients.

Practical considerations:

For all tests of exhaled air and air flow, filters in the expiratory tube should be used to avoid contamination of the room and of the diagnostic devices. This applies to all tests except spiroergometry where this is not suitable for technical reasons.

The instructions for breathing maneuvers should be given by means of microphones and speakers to avoid direct contamination by droplets.

When arterial or arterialized blood gases are taken, both patient and technician should wear a FFP‑2 mask over mouth and nose.

The investigation room and the waiting areas should be well-ventilated to avoid contamination by aerosols.

## Respiratory nursing

Caring for COVID-19 patients and those with suspicion for COVID-19 and their different comorbidities is a big challenge. There have been difficult situations due to outbreaks, room limitation, missing ventilation of the rooms, isolation of patients, false positive and false negative SARS-CoV‑2 diagnostics, and the interference by health authorities with frequently changing regulations.

It belongs to the duties of the nursing staff to adhere to the regulations of the authorities. The aim is to prevent infections of the personnel and infections of other patients. In the clinical setting, nursing staff are used to adhere to high hygiene standards; however, COVID-19 added some new aspects which have otherwise only been used in the care of tuberculosis patients. Infected patients, ideally, should keep a distance of at least 2 m from other people, and wear FFP‑2 masks; however, a number of patients are too sick to wear the mask when the nursing staff directly works on these patients with tight contact.

Therefore, special rules have been implemented by the authorities. The nursing staff must wear a tight FFP‑2 mask over mouth and nose, a face shield, gloves, and a whole-body suit, when patients are approached, even if the nursing staff have been vaccinated or recovered from infection. Hands must be disinfected before putting the gloves on and after taking them off. It has turned out that these protection measures are very exhausting and cannot be sustained for more than 3–4 h per shift. There is no evidence about the protective value of any of these measures.

Only fully trained personel should participate in the care for acute COVID-19 patients. Unfortunately, protection from aerosols by sufficient ventilation of the rooms, where COVID-19 patients are cohorted, is often not sufficient. Technical devices for aerosol clearance (air filter devices or continuous air exchange installations) have often been missing.

The nursing staff are responsible for managing the visitors of patients. There have been very strict regulations, including prohibition of visits or reduction to one visit per patient per day with a maximum stay of 15 min and it may happen that visitors or patients argue with the staff about these regulations or simply do not adhere.

These regulations cause social and emotional isolation and secondary problems for COVID-19 patients. Many patients have difficulties with decisions to make about invasive therapies or diagnostics in a critical situation, they suffer from psychological, social and spiritual stress. These problems are particularly bad if dementia or anxiety disorders is present. Many patients would profit from more intensive contact with their relatives. This causes a dilemma between isolation as necessary measure and its adverse effects. This has been described as the core conflict of the actual situation by German Ethics Council [143]. We recommend that visits are more freely allowed, as long as there is no new local corona outbreak that necessitates strict isolation of the patients.

In the end-of-life situation, in Austrian hospitals, visits have been allowed more freely; however, there is need to better define in which situation the isolation rules are to be lifted. Some guidance for these difficult issues has been provided by Bundesarbeitsgemeinschaft der Seniorenorganisationen (BAGSO) and Dachverband Hospiz Österreich [144–147].

## Cardiopulmonary physiotherapy

Cardiorespiratory physiotherapy interventions include airway clearance techniques, inspiratory muscle training, lung volume recruitment, positioning and early rehabilitation. Referral to physiotherapy interventions in patients with suspected or confirmed acute COVID-19 should be based on an individualized case-by-case analysis in order to avoid unnecessary contact with contagious patients [148–150]. As many cardiorespiratory physiotherapy techniques generate more aerosols than usual, full personal protective equipment and room ventilation is recommended during physiotherapy sessions.

Chest physiotherapy techniques mainly aim at removing airway secretions from the respiratory tract. Patients with mild forms of COVID-19 usually do not develop increased amounts of mucus, but rather present with dry, unproductive cough. There is currently no evidence for prophylactic chest physiotherapy interventions in patients with mild forms of COVID-19 [148]. Routine chest physiotherapy is therefore not indicated in these patients. In patients with certain comorbidities (e.g. COPD, asthma), airway clearance techniques may be necessary. In this case, correct cough and sneezing etiquette should be instructed to the patient prior to starting any respiratory physiotherapy intervention.

Practical considerations:

It has been shown that inhalation with physiologic saline may reduce aerosol production by about 70% for about 1h [8]. Therefore, before the physiotherapist enters the room, the patient should have inhaled physiologic saline for some minutes, possibly with open windows to provide a fully ventilated room with low aerosol concentrations.

Chest physiotherapy should be preferably undertaken with single patient use devices only. If the use of other devices (e.g. mechanical insufflation-exsufflation) is indicated, hygiene precautions and adequate sterilization of those need to be guaranteed at all times.

Respiratory muscle training is not deemed necessary in patients with acute mild forms of COVID-19 as respiratory muscle weakness is not expected in this cohort. Some patients with mild to moderate forms of COVID-19 may be prescribed inhaled corticosteroids or bronchodilators. Physiotherapists may contribute by instructing the patients how to use their inhalers correctly to improve particle deposition.

In patients with severe COVID-19 ARDS, who require admission to the ICU and mechanical ventilation, mucus retention may occur due to prolonged intubation, ineffective cough and bacterial coinfections. In these cases, airway clearance techniques may help in reducing further damage to the lungs. There is currently a lack of evidence on the effectiveness of specific airway clearance techniques in patients with COVID-19. Therefore, no recommendations on which technique to use can be made [149]. Techniques which are highly aerosol-generating should be avoided if possible [150]. Those include all techniques that require disconnection of the ventilator circuit (e.g. manual hyperinflation, mechanical insufflation-exsufflation, positive expiratory pressure).

The routine use of nebulized mucolytic agents (e.g. hypertonic saline) is not recommended, but if necessary, they should be applied by means of closed-circuit nebulizers. Closed-circuit endotracheal suctioning should be used instead of open endotracheal suctioning.

Physiotherapists participate in positioning ICU patients with COVID-19 to optimize matching of the locoregional perfusion to the respective ventilation, improve pulmonary gas exchange and facilitate mechanical ventilation. This includes prone positioning, intermittent lateral positioning and tilting. Prone positioning of awake patients with HFNC or NIV has gathered increased interest since the start of the COVID-19 pandemic [151]. Awake prone positioning can reduce the risk of intubation and should therefore be instructed to the patient, if possible.

Early mobilization and rehabilitation (e.g. sitting on the edge of bed, standing practice, in-bed cycling, neuromuscular electrostimulation) are recommended to reduce the risk of developing ICU-acquired weakness and long-term morbidity (long COVID), especially in patients with prolonged duration of mechanical ventilation [151].
